# Polymorphisms in *CRHR1* and the Serotonin Transporter Loci: Gene × Gene × Environment Interactions on Depressive Symptoms

**DOI:** 10.1002/ajmg.b.31052

**Published:** 2009-12-22

**Authors:** Kerry J Ressler, Bekh Bradley, Kristina B Mercer, Todd C Deveau, Alicia K Smith, Charles F Gillespie, Charles B Nemeroff, Joseph F Cubells, Elisabeth B Binder

**Affiliations:** 1 Department of Psychiatry and Behavioral Sciences, Emory UniversityAtlanta, Georgia; 2 Yerkes National Primate Research CenterAtlanta, Georgia; 3 Howard Hughes Medical InstituteChevy Chase, Maryland; 4 Atlanta VA Medical CenterDecatur, Georgia; 5 Department of Human Genetics, Emory University School of MedicineAtlanta, Georgia; 6 Max-Planck Institute of PsychiatryMunich, Germany

**Keywords:** child abuse, childhood maltreatment, trauma, depression, PTSD, genetic, risk factor

## Abstract

Gene × environment (G × E) interactions mediating depressive symptoms have been separately identified in the stress-sensitive serotonergic (5-HTTLPR) and corticotropin-releasing hormone (CRHR1) systems. Our objective was to examine whether the effects of child abuse are moderated by gene × gene (G × G) interactions between CRHR1 and 5-HTTLPR polymorphisms. We used an association study examining G × G × E interactions of CRHR1 and 5-HTTLPR polymorphisms and measures of child abuse on adult depressive symptomatology. The participant population (N = 1,392) was African-American, of low socioeconomic status (60% with <$1,000/month family income), and with high rates of childhood and lifetime trauma. Depressive symptoms were measured with Beck Depression Inventory (BDI) and history of Major Depression by Structure Clinical Interview based on DSM-IV (SCID). We first replicated an interaction of child abuse and 5-HTTLPR on lifetime SCID diagnosis of major depression in a subsample (N = 236) of the study population—the largest African-American 5-HTTLPR cohort reported to date. We then extended our previously reported interaction with both a CRHR1 SNP (rs110402) and TCA haplotype interacting with child abuse to predict current symptoms (N = 1,059; *P* = 0.0089). We found that the 5-HTTLPR S allele interacted with CRHR1 haplotypes and child abuse to predict current depressive symptoms (N = 856, *P* = 0.016). These data suggest that G × E interactions predictive of depressive symptoms may be differentially sensitive to levels of childhood trauma, and the effects of child abuse are moderated by genetic variation at both the CRHR1 and 5-HTTLPR loci and by their G × G interaction. © 2009 Wiley-Liss, Inc.

## INTRODUCTION

Epidemiological and molecular genetic data have established that both environmental and genetic factors contribute to the risk of major depression, with heritability estimates ranging between 30% and 40%, with the remaining variance influenced by mostly individual specific environmental factors [Kendler, 1995; Levinson, 2006]. Twin and family studies have suggested that the genetic contribution is most likely oligo- to polygenetic, with many genetic variations of smaller effect contributing in an additive or interactive manner [Levinson, 2006]. Recently, two large genome-wide association studies for unipolar depression have been published, which reported more or less negative results for larger main genetic effects [Muglia et al., 2008; Sullivan et al., 2008]. Both studies were case–control associations, and neither included environmental exposure as a factor nor investigated gene × gene (G × G) interactions. Inclusion of gene × environment (G × E) and G × G interactions in such analyses might, however, be critical to detect genetic effects in unipolar depression. Genome-wide G × E or gene × gene × environment (G × G × E) studies for unipolar depression have, to the best of our knowledge, not yet been conducted and would require very large sample sizes to attain sufficient power. In this study, we therefore focus on a candidate G × G × E interaction.

The most established environmental risk factors for major depression are stressful life events, with early exposure to trauma being a particularly strong risk factor in conferring risk for the development of depression. Specifically, a history of adverse childhood experience (e.g., child abuse or early separation from parents) increases the risk for developing major depression as an adult by two- to threefold, and there is a highly significant dose–response relationship between the degree of early adverse experience and depressive disorders as adults [Chapman et al., 2004; Anda et al., 2006; Bradley et al., 2008]. Early trauma has been associated with long-lasting abnormalities in several biological systems that have also directly been implicated in the pathophysiology of major depression, including the serotonergic (5HT) and the corticotropin-releasing factor (CRF) systems [Arborelius et al., 1999; Heim and Nemeroff, 2002; Edwards et al., 2003; Nemeroff, 2004].

In fact, both rodents and non-human primates exposed to early adverse experiences exhibit evidence of hyperactivity of the CRF system as adults, as measured by cerebrospinal fluid (CSF) CRF concentrations, CRF and CRF receptor type 1 (CRF1) mRNA expression, and CRF receptor binding [Plotsky and Meaney, 1993; Coplan et al., 1996; Ladd et al., 2000; Plotsky et al., 2005]. These observations in laboratory animals are consistent with the findings in humans where early life trauma is a strong predictor of CSF CRF concentration in adults [Carpenter et al., 2004; Lee et al., 2005, 2006]. It is also associated with an enhanced stress response to psychosocial stressors, such as the Trier Social Stress Test, as well as to endocrine challenge tests, including the CRF stimulation test and the combined dexamethasone suppression/CRF stimulation test [Heim et al., 2000, 2001, 2008; Tyrka et al., 2008].

In rodents, maternal separation in the early postnatal period also leads to persistent alterations in serotonergic systems, including changes in cell firing of serotonergic raphe neurons, frontal cortical 5-HT concentration, and expression levels of certain serotonin receptors in cortex and hippocampus [Arborelius et al., 1999; Matthews et al., 2001; Gardner et al., 2005; Vicentic et al., 2006; Arborelius and Eklund, 2007; Lambas-Senas et al., 2009]. In primates, stressful rearing conditions are associated with altered CSF concentrations of the major serotonin metabolite 5-hydroxyindoleacetic acid (5-HIAA) [Mathew et al., 2002] and in humans, child abuse was shown to negatively correlate with CSF 5-HIAA concentrations [Roy, 2002] and to be associated with dysregulation in serotonergic function [Tiihonen et al., 1997; Rinne et al., 2000; Steiger et al., 2001].

The fact that early adverse experience impacts both CRF and 5-HT systems is likely due, in part, to the strong interconnection between the two neural circuits. CRF has been reported to inhibit serotonergic neurons in the dorsal raphe nuclei [Price et al., 1998; Kirby et al., 2000] and may thus modulate serotonin release in terminal areas [Price and Lucki, 2001]. Very recent data suggest that CRF leads to inhibition of raphe 5-HT neurons via increased inhibitory action of GABA [Kirby et al., 2008]. Brain regions central to the stress response such as the hippocampus and prefrontal cortex in turn receive dense serotonergic innervation, and serotonin release from the raphe leads to inhibition within the basolateral amygdala [Rainnie, 1999].

Maternal deprivation in rats is associated with an increase in CRF binding sites in the raphe nuclei [Ladd et al., 1996]. In primates exposed to early adverse environment, there is a negative correlation between CSF CRF and 5-HIAA concentrations [Mathew et al., 2002], supporting an interacting effect of these two systems in the biology of early adverse experience. Consistent with these data, a number of studies have demonstrated that serotonin reuptake inhibitor (SRI) antidepressants reverse the stress-related effects of CRF administration [Lowry et al., 2008], and human depressed patients treated with SRIs show decreased concentrations of CSF CRF [De Bellis et al., 1993; Nikisch et al., 2005]. Furthermore, dysregulation in both systems has been implicated in the pathophysiology of major depression. Child abuse-induced alteration of these systems could thus be causally involved in the increased risk for depression conferred by early life trauma.

In light of these data, it is thus not surprising that genetic variants impacting the function of genes within these systems have been shown to moderate the effects of early trauma on depression. The serotonin transporter-linked polymorphic region [Lesch et al., 1996; Caspi et al., 2003; Eley et al., 2004; Gillespie et al., 2005; Grabe et al., 2005; Kendler et al., 2005; Munafo et al., 2008; Surtees et al., 2006; Taylor et al., 2006; Uher, 2008] (5-HTTLPR) has repeatedly been shown to interact with life stress and childhood maltreatment to predict adult depression. While a meta-analysis could not validate this interaction for number of life events [Risch et al., 2009], no meta-analysis so far has been reported for the interaction with childhood maltreatment. A number of studies report a significant moderation of the effects of childhood maltreatment on adult depressive or psychiatric disorders [Caspi et al., 2003; Kaufman et al., 2004; Gibb et al., 2006; Roy et al., 2007; Richardson et al., 2008], in that the short allele of this polymorphism is associated with an increase in symptom severity with early trauma severity. Interaction of the 5-HTTLPR may thus be more robust with early trauma than with life events in general. We have recently reported an interaction of child abuse with polymorphisms in the type 1 CRF receptor gene, *CRHR1*, to predict adult depression in two independent cohorts [Bradley et al., 2008]. This finding has now been replicated in an independent, Caucasian cohort [Polanczyk et al., 2009], supporting the trans-ethnic relevance of the investigated interaction. This G × E interaction has also been shown to have endocrine repercussions, with the CRHR1 polymorphisms moderating childhood maltreatment-related HPA-axis hyperactivity [Tyrka et al., 2009].

Because of the above-described interaction between these two systems on a biological level, it is also possible that G × G interactions across these systems moderate the impact of early adverse experience. In fact, such G × G × E interactions have been reported for the *BDNF* Val66Met polymorphism as well as a functional interleukin-6 (IL6) polymorphism with the *5-HTTLPR*, for example [Kaufman et al., 2006; Kim et al., 2007; Bull et al., 2008; Wichers et al., 2008]. The aim of the current study was to examine the combined interaction of the 5-HTTLPR and *CRHR1* polymorphisms with childhood abuse in predicting current symptoms of depression in a sample of over 1,000 African-American men and women.

## METHODS

### Sample and Sample Recruitment

The data from this study were collected as part of a larger study investigating the roles of genetic and environmental factors in predicting response to stressful life events in a predominantly African-American, urban population of low socioeconomic status (SES). This is an expanded study from our original publication demonstrating *CRHR1* polymorphisms × child abuse interactions with depression [Bradley et al., 2008]. Research participants were approached while either waiting for their medical appointments or while waiting with others who were scheduled for medical appointments, in the waiting rooms of the primary care clinic or obstetrical–gynecological clinic of a large urban, public hospital. Subjects who indicated willingness to participate provided written informed consent, participated in a verbal interview, and provided a salivary sample for DNA extraction (described below). The data presented in this article are from the first 1,492 subjects interviewed of which only data from the 1,392 individuals self-identifying as African-American were used in this report (93.3%) to decrease any potential confounds due to ethnic stratifications. All procedures in this study were approved by the Institutional Review Boards of Emory University School of Medicine and Grady Memorial Hospital. Demographic data on gender, self-identified race/ethnicity, education, employment, disability status, and monthly household income are presented in Table I.

**TABLE I:** Sample Demographics

Demographic (*all self-identified African-American/Black*)	Total sample, N (%)	Male, N (%)	Female, N (%)
Age (N = 1,385)			
18–24	260 (19)	58 (11)	198 (23)
25–34	261 (19)	74 (14)	186 (22)
35–44	250 (18)	99 (19)	149 (17)
45–54	377 (27)	182 (35)	193 (23)
55–64	185 (13)	86 (17)	98 (11)
≥65	52 (4)	21 (4)	31 (4)
Education (N = 1,378)			
<12th grade	354 (26)	127 (24)	227 (26)
High school graduate	527 (38)	201 (39)	326 (38)
Graduate equivalency diploma	71 (5)	27 (5)	44 (5)
Some college/technical school	274 (20)	100 (19)	174 (20)
Technical school graduate	52 (4)	16 (3)	36 (4)
College graduate	82 (6)	39 (8)	43 (5)
Graduate school	18 (1)	10 (2)	8 (1)
Employment (N = 1,380)			
Currently unemployed	912 (66)	358 (69)	554 (65)
Currently employed	468 (34)	164 (31)	304 (35)
Disability (N = 1,374)			
Currently receiving disability	308 (22)	142 (27)	166 (19)
Not receiving disability	1,066 (78)	376 (73)	690 (81)
Household monthly income in US$ (N = 1,351)			
0–499	508 (38)	199 (38)	309 (37)
500–999	363 (27)	134 (26)	229 (27)
1,000–1,999	331 (25)	118 (23)	213 (26)
≥2,000	149 (11)	66 (13)	83 (10)

### Procedure

Participants verbally completed a battery of self-report measures, including the Beck Depression Inventory (BDI) and the brief Child Trauma Questionnaire, which took 45–75 min to complete (dependent in large part on the extent of the participant's trauma history and symptoms). We read the instruments to participants to guard against relatively high rates of impaired literacy. Each person was paid $15.00 for participation in this phase of the study. The measures used in the current study are described below.

#### Beck Depression Inventory (BDI)

Depressed mood was assessed with the 21-item BDI [Beck et al., 1961], a well-validated, commonly used continuous measure of level of depressive symptoms. In this sample, the BDI had a standardized alpha coefficient of 0.99 (M = 14.4, SD = 13.2).

Additionally, because these analyses were performed in subjects identified in a general medical clinic, it is possible that the BDI is a proxy for medical-related depressive symptomatology separate from Major Depressive Disorder (MDD). To examine this, we have previously used history of prescribed medicine as a proxy variable for medical illness category [Bradley et al., 2008]. There were no significant effects found for any of the 13 available classes of medicines examined (*P*'s > 0.1). This demonstrates that the moderation effects of child abuse and *CRHR1* genotype were unlikely to be affecting medical condition as a possible contributor to depressive symptoms.

#### Structured Clinical Interview according to DSMIV (SCID)

Two hundred ninety-seven individuals underwent more extensive psychopathological evaluations including the Structured Clinical Interview for DSM-IV (SCID-DSMIV [First et al., 1995]). This is a validated interview assessment of DSM-IV mood disorders and was used to assess the presence or absence of MDD as well as other mood disorders within our study population.

#### Childhood Trauma Questionnaire (CTQ)

The CTQ [Bernstein and Fink, 1998] is a self-report inventory assessing three types of childhood abuse: sexual, physical, and emotional. Studies have established the internal consistency, stability over time, and criterion validity of both the original 70-item CTQ and the current brief version [Bernstein et al., 2003]. The CTQ yields a total score and subscale scores for each of the types of child abuse. Our CTQ data demonstrated high internal reliability (α = 0.99 for physical abuse; 0.94 for sexual abuse; 0.93 for emotional abuse; 0.98 for the total of these three scales). Bernstein and Fink 1998 established scores for none, mild, moderate, and severe for each type of abuse. The data from the CTQ were used to classify participants into two categories for each type of abuse (physical, sexual, emotional). For each type of abuse (sexual, physical, and emotional) those participants with CTQ scale scores in the none-to-mild range were classified as negative/absent and those with CTQ scores in the moderate-to-severe range were classified as positive/present. We then created a composite variable across all three types of abuse. We first created a two-level abuse variable dividing participants into two groups with respect to numbers of types of abuse on which they fell into the moderate-to-severe range: (1) those with no type of abuse in the moderate-to-severe range and (2) those with moderate-to-severe on at least one type of abuse. We also created a three-level abuse variable: (1) those with no type of abuse in the moderate-to-severe range, (2) those with moderate-to-severe abuse in one type of abuse, and (3) those with moderate-to-severe abuse in two or more types of abuse.

### DNA Extraction

DNA was extracted from saliva collected into Scope mouthwash (N = 46) or into Oragene saliva kits (DNAGenotek, Inc., Ontario, Canada) for the rest of the sample using the Qiagen M48 system and the Purelink 96 Genomic DNA Kit from Invitrogen (Carlsbad, California) (Cat # K1821-04). High-quality DNA was available for 1,234 individuals. The 158 individuals with no genotype information consist of individuals for which DNA was not collected or for which we attempted to collect DNA using Oragene-saliva samples but DNA extraction failed completely in two separate extraction trials (these failures were either due to non-compliance of the study participants or failure to correctly break the seal that releases the stabilizing solution in the Oragene kits). When comparing the individuals with genotypes to those without, we found no significant difference on any parameters relevant for the study as described before in Binder et al. 2008.

### Single-Nucleotide Polymorphisms (SNP) and Genotyping

The genetic methodology for SNP genotyping is described more extensively in our previous publications from this sample [Binder et al., 2008; Bradley et al., 2008]. We examined the *CRHR1* SNPs (rs7209436, rs4792887, and rs110402) and the resulting haplotypes based on our previous examination of this gene and depression [Bradley et al., 2008]. All SNPs were genotyped using a TaqMan allelic discrimination assay [Livak, 1999] developed for use on the 7900HT instrument (Applied Biosystems, Foster City, CA) using predesigned and validated TaqMan assay reagent kits containing one pair of PCR primers and one pair of fluorescently labeled probes (Applied Biosystems; http://www.appliedbiosystems.com/). PCRs were performed in 5 µl reaction volumes in 384-well plates and contained 5 ng of DNA. The standard protocol provided with the kit was followed. Thermal cycler conditions were 95°C for 10 min, 40 cycles of 95°C for 15 sec and 60°C for 1 min. The SDS 2.3 software was used for allelic discrimination. For quality control, 45% of the samples were genotyped as duplicates across and within a 384-well plate. Ten discordances were recorded for 4,685 non-duplicate genotypes (0.20%) and excluded from the analysis. Call rates for the three SNPs ranged from 90.7% to 99.2%. All assay IDs, SNP position, and Hardy–Weinberg equilibrium are *available on request from the authors*.

Genotyping of the 5-HTTLPR utilized the following primers (forward: 5′-Hex-TGAATGCCAGCACCTAACC-3′; reverse: 5′-ATACTGCGAGGGGTGCAG-3′). PCR was carried out in 384-well plates in a 10 µl volume with 10 ng DNA. Each PCR reaction contained 0.5 µM of each primer, 0.08 µM of dATP, dCTP, and dTTP, and 0.04 µM of dGTP, 0.2 µM of 7-deaza GTP (Amersham Biosciences/GE Healthcare, Piscataway, NJ), 5% DMSO, and 1.25 U of AmpliTaq Gold (Applied Biosystems). The cycling parameters were as follows: 95°C for 5 min, then 94°C for 30 sec, 63°C for 30 sec, and 72°C for 1 min for 1 cycle, then the annealing temperature reduced to 62°C for one more cycles and then to 59.5°C for 38 cycles. Five microliters of the resulting PCR products was then digested with 5 U *Msp*I (New England Biolabs) in a total volume of 10 µl for 90 min at 37°C to detect the A/G SNP rs25531 shown to influence the functional effects of the long and short alleles [Hu et al., 2006]. The digested PCR products were then separated using an Applied Biosystems 3100 genetic analyzer and analyzed with Applied Biosystems Genemapper 4.0 software. Fragment lengths for the L-A allele are 291 bp, 148 for the L-G, and 247 bp for the S allele. The VL fragment was 335 bp and the XL fragment was 375 bp.

We genotyped 506 samples in duplicates and out of these only 3 discordant genotypes were observed and the call rate was 94.6%.

### N in Analysis

Of the 1,234 samples with sufficient DNA, all entered *CRHR1* SNP genotyping and 1,133 entered 5-HTTLPR genotyping. Of the 1,392 total samples, 1,213 had a valid entry for both the BDI as well as the CTQ. Of these, all 1,213 had attempted *CRHR1* genotyping, 990 had attempted 5-HTTLPR genotyping, and 964 had overlapping *CRHR1* and 5-HTTLPR genotyping. Due to less than 100% call rates the final N in the G × E analyses was smaller than the attempted genotypes and is specified for each analysis.

### Variable Coding and Regression and Permutation Analyses for Interaction Effects

We used linear regression to assess whether the genotypes itself or in interaction with child abuse predicted current depressive symptoms. Predictors: 5-HTTLPR was coded as SS, SL, LL, extra-long alleles (VL and XL), or as S-allele carriers or using functional classification as previously described [Hu et al., 2006]. In the latter, low function genotypes are SS, SLG, and LGLG. Intermediate function alleles are SLA and LALG, and the high function genotype is LALA. In this classification very or extra-long allele carriers were excluded as were for the S-allele carrier status. For *CRHR1* variants, we investigated the effects of the best SNP from Bradley et al. 2008, rs110402 using a protective allele carrier model (AA and AG vs. GG—see Table II) and a carrier model of the protective haplotype described in this article. The SNPs rs7209436, rs4792887, and rs110402 formed three major haplotypes—CCG with 35.3%, CTG with 32.9%, and TCA with 30.4%. All other haplotypes were less than 1% in this sample. In our previous study, we had identified the TCA haplotype as protective, so that the absence of any TCA haplotypes was coded as *CRHR1* risk = 1, and the presence of at least one copy of this haplotype as *CRHR1* risk = 0 (see Table II). Child abuse was coded into the two (none-to-mild and moderate-to-severe overall) or three levels (no moderate or severe type of abuse, one type of moderate or severe abuse of two types) described above. We further adjusted all regression models for potential confounders by including age and gender. For linear regression, we additionally examined the significance of genotype–child abuse interaction effects using *permutation-based* procedures [Li et al., 2000; Epstein and Satten, 2003] that randomly assigned the sample BDI scores to subjects (sampled without replacement), while holding each subject's genotype and or haplotypes and environmental variables fixed. This permutation method is robust against non-normal distribution of the outcome variable as we observe with the BDI in this population [Bradley et al., 2008]. For each analysis, the empirical *P*-value was based on 10,000 permutations. We conducted these analyses using appropriate components of the SAS software system (version 9.1, SAS Institute, Cary, NC). Haplotypes were estimated using SNPHAP [Adkins, 2004; Sham et al., 2004]. All haplotypes with an estimation probability less than 95% were excluded from the analysis (4.1% excluded). For effects on SCID-based lifetime diagnosis, we used logistic regression in a subsample (N = 236–278) using 5-HTTLPR or *CRHR1* rs11402 A-carrier status and the two-level child abuse variable and their interaction terms as predictors, adjusting for age and sex. Due to the much smaller N with available SCID data, we did not analyze G × G × E interaction analyses for this outcome.

**TABLE II:** Frequency of 5-HTTLPR and *CRHR1* Genotypes

5-HTTLPR genotype			5-HTTLPR genotype and rs25531			
		
			Functional classification [Hu et al., 2006]			
				
	N	%		N	%	
LL	598	55.78	LALA	292	27.2	High
			LALG	269	25.1	Intermediate
			LGLG	37	3.5	Low
SL	359	33.49	SLA	243	22.7	Intermediate
			SLG	116	10.8	Low
SS	66	6.16		66	6.2	Low
LXL	35	3.26	LAXL	20	1.9	
			LGXL	15	1.4	
SXL	8	0.75		8	0.8	
LVL	6	0.56	LAVL	3	0.3	
			LGVL	3	0.3	
N total	1,072			1,072		

*CRHR1* rs110402			*CRHR1* TCA haplotype		
	
	N	%		N	%
AA	87	7.2	0 allele	577	48.8
AG	544	44.8	1 allele	519	43.9
GG	582	48.0	2 alleles	87	7.3
N total	1,234				

SERT*TCA haplotype risk		

	N	%
No risk alleles	286	24.2
S-allele only	214	18.1
CRHR1 risk only	293	24.8
Both risk alleles	195	16.5

## RESULTS

### Frequency of 5-HTTLPR Genotypes in African-American Sample

As shown in Table II, the frequency of the 5-HTTLPR within this sample is 6.2% SS, 33.5% SL, and 55.8% LL with 4.7% carrying a longer form such as the VL-17 repeats (LVL 0.6%) and the XL—18 repeats (LXL = 3.3%, SXL = 0.7%) genotypes. For rs25531, the frequency of G-allele carriers in individuals carrying at least one L allele was 43.9% and using the functional classification proposed by Hu et al. 2006, 21.4% were low functioning, 50.0% intermediate function, and 28.6% high function genotype carriers (see Table II).

### 5-HTTLPR Genotype, Child Abuse, and Current Adult Depression Symptoms

**FIG. 1:**
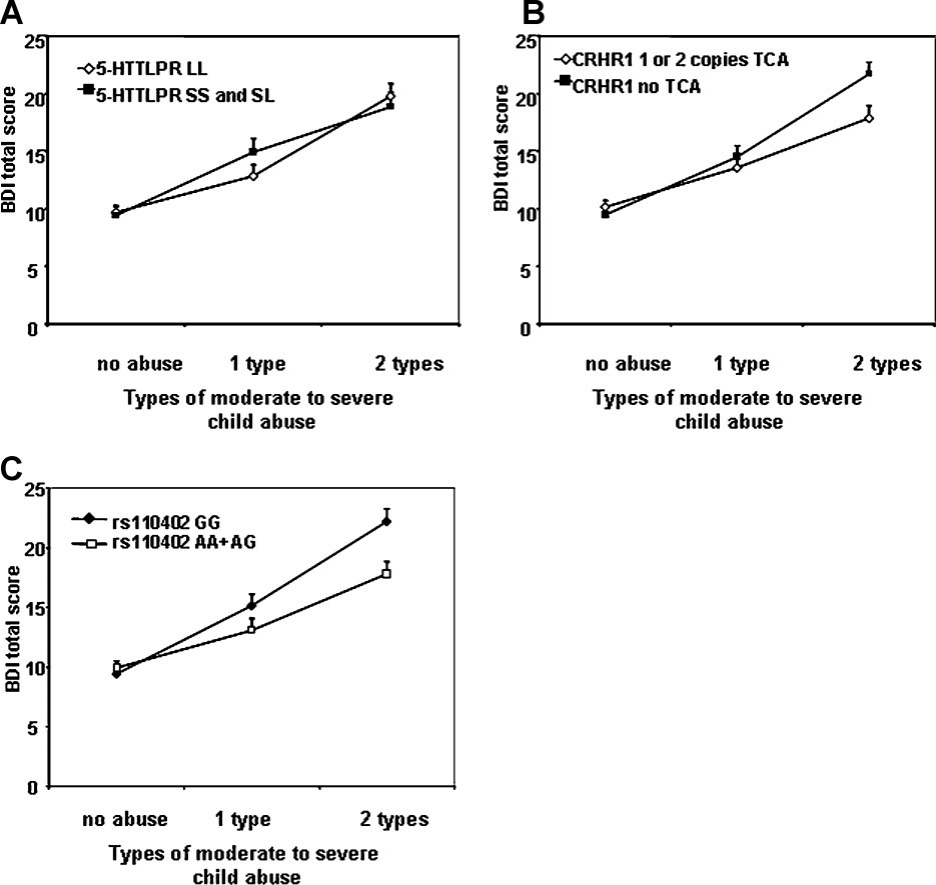
Interaction of 5-HTTLLPR S-allele carrier status and CRHR1 TCA haplotype with child abuse to predict adult depression. **A**: Depression symptoms using the Beck Depression Inventory (BDI) are graphed as a function of 5-HTTLPR SS, SL versus LL carrier status, as a function of level of moderate-to-severe child abuse. We find no significant main or interaction effects. **B**: Depression symptoms using the BDI are graphed as a function of TCA haplotype (rs7209436, rs4792887, and rs110402) of the *CRHR1* gene, as a function of level of moderate-to-severe child abuse, demonstrating a significant interaction effect (*P* < 0.05). **C**: Depression symptoms using the BDI are graphed as a function of *CRHR1* rs110402 (A-carrier model), as a function of level of moderate-to-severe child abuse, demonstrating a significant interaction effect (*P* = 0.009).

We first evaluated whether the 5-HTTLPR genotype, S-allele carrier status, or functional classification together with child abuse (two- or three-level category) would predict current depressive symptoms, using the continuous total score from the BDI as outcome, and co-varying for sex and age (N = 926 for all genotypes and N = 885 for S-allele carrier status and functional classification). We found that although there was a highly significant effect of level of child abuse history (two-level) on BDI (*P* < 0.0001), there was no significant main effect of the 5-HTTLPR nor interaction between the 5-HTTLPR and two-level child abuse on BDI total score, for all types of 5-HTTLPRs coding. The same outcome was observed when using a three-level categorization of child abuse (Fig. 1A, 5-HTTLPR S-allele carrier status).

### 5-HTTLPR Genotype, Child Abuse, and Lifetime History of Major Depression

We then tested the interaction of the 5-HTTLPR genotype, S-allele carrier status, and 5-HTTLPR functional classification with child abuse (none to mild vs. moderate to severe) to predict lifetime diagnosis of depression as measured with SCID-DSMIV, with age and sex as co-variates (N = 236). In these analyses, childhood abuse (*P* < 0.0001, B ± SD = 2.21 ± 0.59) as well as its interaction term with the 5-HTTLPR genotype (*P* = 0.016, B = −0.82 ± 0.33) were included in the model. When using the S-allele carrier status, only its interaction term with child abuse remained a significant predictor in the model (*P* = 0.00017, B = 1.36 ± 0.36). As previously reported [Caspi et al., 2003], we also see an over-representation of S-allele carriers in the group with child abuse and lifetime major depression (see Table III). No significant 5-HTTLPR × child abuse interaction was observed using the functional allele classification.

**TABLE III:** Distribution of 5-HTTLPR Genotype by Child Abuse and Lifetime History of Major Depression Diagnosed With SCID-DSMIV (Total N = 236)

	5-HTTLPR genotype			N total
	
	SS	SL	LL	
No to mild abuse				
No lifetime MDD				
N	6	30	62	100
%	6.0	30.0	64.0	
Lifetime MDD				
N	1	13	21	35
%	2.9	37.1	60.0	
Moderate to severe abuse				
No lifetime MDD				
N	2	13	37	52
%	3.8	25.0	71.2	
Lifetime MDD				
N	6	20	23	49
%	12.2	40.8	46.9	

The S-allele carrier group is the combination of the cells from SS and SL.

### CRHR1, Child Abuse, and Current Adult Depression Symptoms

In this analysis, we first re-examined our previously reported finding of *CRHR1* genotype interaction with level of child abuse to predict adult depression with the most significant SNP rs110402 and the TCA haplotype previously described [Bradley et al., 2008]. The analysis was performed using both the two- and three-level child abuse variables. The N for this G × E interaction in the current study is more than double (N = 1,059) our prior study (N ∼ 480), with the former sample pool included in the current study. We find a significant G × E interaction (co-varying for age and sex) with the two-level child abuse variable with the A allele of rs110402 (B = 3.53 ± 1.35, *P* = 0.0089, empirical *P* = 0.0095) and the presence of the protective *CRHR1*-TCA haplotype (B = 1.59 ± 1.66, *P* = 0.039) but no genetic main effects. Similar results were observed for the three-level child abuse variable with a significant interaction of child abuse and rs110402 A carrier status (B = 4.88 ± 1.7, *P* = 0.012, empirical *P* = 0.011) and a main effect *CRHR1* A carrier status (B = 4.38 ± 1.5, *P* = 0.0071) as well as a significant interaction of TCA risk haplotype carriers with child abuse (B = 1.86 ± 0.85, *P* = 0.033) (see Fig. 1B,C). In all models, child abuse (both as the two-level as well as three-level variable) was a highly significant predictor (*P* < 0.0001), as were sex and age (*P* < 0.05 in all models).

**FIG. 2:**
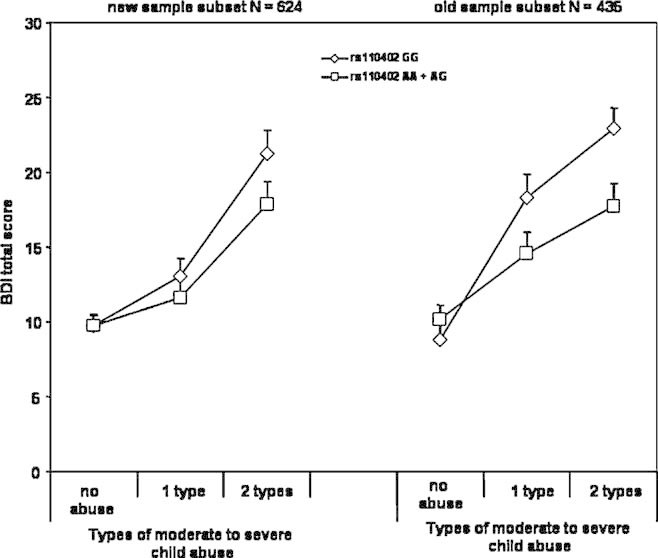
Interaction of *CRHR1* rs110402 with replicate samples. Depression symptoms using the BDI are graphed as a function of the*CRHR1* rs110402 SNP, GG versus AA, AG as a function of level of moderate-to-severe child abuse in the sample reported in Bradley et al. 2008 (old) and additional samples reported in this manuscript (new).

Because data from a fraction of this sample (N = 435) had been previously reported, we investigated whether similar G × E interactions were observed in the first and the second data sets that comprise the present sample. We added “presence in the first report” coded as 0 or 1 as a predictor in the analysis in addition to its interaction term with child abuse and with child abuse × rs110402 A carrier status. We re-reran the analyses with both the two-level and the three-level abuse categorizations. In both analyses, we found a significant main effect of subsample on BDI total score and interaction between child abuse and presence in first article (*P* < 0.05) in addition to age, sex, and child abuse. No analyses indicated a significant presence in analysis × rs110402 × child abuse interaction, indicating that the interaction effect was not different in the two sets of samples (see Fig. 2). The two sample subsets had, however, significant differences in environmental exposure and depression scores, possibly explaining slight differences in the interaction. The latter samples had significantly lower BDI total scores (11.05 ± 10.5 (SD) vs. 13.5 ± 12.2 (SD), *F*_1233,1_ = 8.6, *P* = 0.003), lower CTQ total scores (38.9 ± 15.4 vs. 44.2 ± 17.2, *F*_1297,1_ = 32.2, *P* < 0.001) and were older (41.2 ± 13.9 years vs. 39.2 ± 14.2 years). There was no difference in gender distribution (62.3% female vs. 62.9%, *P* = 0.81) and *CRHR1* genotype distribution (36.9% rs110402 A allele carriers vs. 38.7%, *P* = 0.53).

### CRHR1, Child Abuse, and Lifetime History of Major Depression

We then tested the interaction of the rs110402 A carrier status with child abuse (none to mild vs. moderate to severe) to predict lifetime diagnosis of depression as measured with SCID-DSMIV, with age and sex as co-variates (N = 275). In these analyses only childhood abuse (*P* = 0.00022) was included in the model. While rs110402 carrier status and its interaction term were not significant, we found a nominal over-representation of protective A-allele carriers in the abuse but not major depression group (see Table IV).

**TABLE IV:** Distribution of rs110402 A-Allele Carriers by Child Abuse and Lifetime History of Major Depression Diagnosed With SCID-DSMIV (Total N = 278)

	rs110402, GG	rs110402, AA + AG	Total N/group
No to mild abuse			
No lifetime MDD			
N	59	60	119
%	49.6	50.4	
Lifetime MDD			
N	22	19	41
%	53.7	46.3	
Moderate or severe abuse			
No lifetime MDD			
N	25	36	61
%	41.0	59.0	
Lifetime MDD			
N	30	27	57
%	52.6	47.4	

### 5HTLLPR, CRHR1, Child Abuse, and Current Adult Depression Symptoms

We next examined the data for a G × G × E interaction between the 5HTLLPR (S-allele carrier) and *CRHR1* haplotype (protective haplotype carrier) and level of child abuse history. Given the number of predictors in the model with the 856 subjects for whom we have all genotype and phenotype data, we found that we would have >95% power to detect a *P*-value at 0.01 that accounts for more than 5% of the variance in a G × G × E model.

**FIG. 3:**
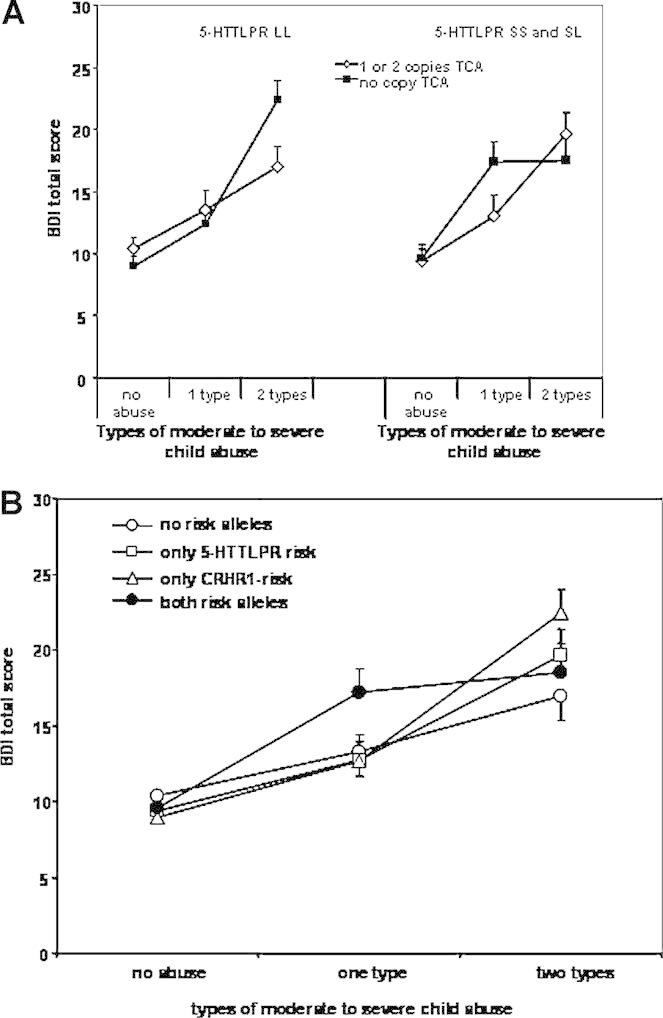
Interaction between 5-HTTLPR S-allele carrier status and *CRHR1* TCA haplotype carriers and child abuse on adult depressive symptoms. **Panel A**: Depression symptoms using the BDI are graphed as a function of *5-HTTLPR* and *CRHR1* genotype. **Left panel**: Individuals with 5-HTTLPR LL genotypes are represented with lines representing one or copies versus no copies of the protective TCA *CRHR1* haplotype. **Right panel**: Individuals with 5-HTTLPR SS or SL genotypes are represented with lines representing one or copies versus no copies of the protective TCA *CRHR1* haplotype. An overall gene × gene × environment interaction is seen (N = 856, *P* = .016). **Panel B**: Representation of the same interaction but grouping the individuals by number of risk variants. No abuse group: N = 159 no risk alleles, N = 115 only 5-HTTLPR s-allele, N = 148 only CRHR1 risk, N = 102 both risk alleles. One type of abuse group: N = 44 no risk alleles, N = 39 only 5-HTTLPR s-allele, N = 69 only *CRHR1* risk, N = 44 both risk alleles. Two types of abuse: N = 42 no risk alleles, N = 36 only 5-HTTLPR s-allele, N = 46 only *CRHR1* risk, and N = 30 both risk alleles.

Although no significant G × G × E interactions were observed with the two-level child abuse variable, we find a significant G × G × E interaction (co-varying for age and sex) when using the three-level child abuse variable. In this model, we observe significant effects of age, sex, and child abuse level (all *P*-values < 0.05), as well as a significant G × G × E interaction (N = 856, B = 9.26 ± 3.95, *P* = 0.0161, empirical 0.0164; see Fig. 3A). Note that after log transforming the BDI score to account for any possible skewness, the G × G × E interaction remains (*P* < 0.01). The protective effect of the *CRHR1* TCA haplotype is only seen in absence of the 5-HTTLPR S allele (Fig. 3, left panel) and only in the absence of the TCA protective haplotype, the S allele increases current depression with moderate levels of child abuse (Fig. 3A, right panel). Using a combined coding of presence of risk alleles (no risk group = 5-HTTLPR LL and one or two copies of *CRHR1* TCA; *CRHR1* risk only = 5-HTTLPR LL and 0 copies *CRHR1* TCA; 5-HTTLPR risk only = 5-HTTLPR SS or SL and one or two copies *CRHR1* TCA; and combined risk group = 5-HTTLPR SS or SL and 0 copies *CRHR1* TCA), lower levels of child abuse severity are sufficient to elicit clinically relevant symptoms of depression in individuals with both genetic risk factors, as compared to the no risk or single genetic risk groups (see Fig. 3B).

## DISCUSSION

This article presents three primary findings. First, we did not find a main effect of 5-HTTPLR genotype or an interaction effect of 5-HTTPLR and child abuse on current level of depression symptoms in a sample of over 900 African-American men and women but did replicate an interaction of child abuse and 5-HTTLPR on lifetime diagnosis of major depression in a subsample of 234 individuals. Second, using a data set of 624 more individuals, we extended our previously reported interaction of CRHR1 variants with child abuse to predict adult depressive symptoms in a sample of now over 1,000 individuals. Third, we found that the 5-HTTLPR interacts with CRHR1 variants and child abuse to predict current, adult depressive symptoms: individuals carrying the risk alleles in both genes exhibit clinically relevant depressive symptoms at less severe levels of child abuse than individuals with no or only one of the risk alleles.

To the best of our knowledge this the first article to present data on 5-HTTPLR and depressive symptoms in an African-American sample—though a previous study in a similar population investigated this polymorphism as predictor of suicide attempts in a sample with a history of drug abuse [Roy et al., 2007]. The observed allele frequencies for the 5-HTTLPR, including the VL and XL alleles, are in agreement with previous publications and support that the SS genotype (6–7%) is much less frequent in individuals from African-American descent than in European Americans (20–25%) [Gelernter et al., 1997, 1998; Hu et al., 2006]. The XL allele has thus far only been observed in individuals from African descent and VL allele had previously been detected in Asian populations [Gelernter et al., 1997; Nakamura et al., 2000]. The few VL alleles observed in this sample might be related to admixture from native American or Asian ancestry that we observe in our sample [Bradley et al., 2008].

Consistent with the majority of the extant literature, we did not find a main effect of 5-HTTLPR genotype on current symptoms of depression in this sample. We replicate the previously described increased prevalence of incidence of lifetime major depression in individuals carrying the 5-HTTLPR S allele and exposed to child abuse [Caspi et al., 2003], but this interaction was not seen when using the functional classification of Hu et al. 2006. Possibly, the higher prevalence of the low-function rs25531 G allele in African-American compared to Caucasians as well as additional genetic differences of this locus depending on ethnic background might lead to this discrepancy. We, however, failed to find a significant interaction of childhood abuse and 5-HTTLPR on current depression symptoms in this sample. To resolve the conflicting data on lifetime versus current depression for this G × E interaction in our sample, we are currently gathering more comprehensive data on factors such as age of onset of abuse, age of onset, and recurrence of depression symptoms in the current sample which will allow us to more closely analyze the roles of these factors in the current sample. While a recent meta-analysis concluded that the observed interaction of the 5-HTTLPR with life events to predict adult depression is likely attributable to chance [Risch et al., 2009], these controversial results might be due to sample-related differences in such factors including age, gender, type of environmental exposure (early life trauma vs. recent life stressors), and type of depression (recent onset adult vs. chronic) [Brown and Harris, 2008].

In a sample expanded to 1,059 individuals, we were able to extend our previously reported interaction of *CRHR1* variants and child abuse to predict adult depressive symptoms [Bradley et al., 2008]. Using more graded levels of child abuse as the environmental variables, we could show that the protective effects of *CRHR1* variants are more pronounced with the presence of at least one type of severe abuse. However, it is important to note, as in Figure 3, that sufficiently high levels of environmental exposure to trauma may overcome any genetic effects. The additional samples not reported in the initial manuscript show an interaction of *CRHR1* variants and child abuse in the same direction, but less pronounced than in the first sample. This might be due to differences of the initial sample versus the second set of recruited individuals. The more recently recruited individuals had lower BDI and CTQ scores and were on average 2 years older, while gender and *CRHR1* haplotype and 5-HTTLPR genotype distribution was the same. These differences in environmental exposure and depressive symptoms might, in part, explain the differences in effect size between the first and second set of samples (see Fig. 2). We did not see a significant interaction of *CRHR1* variants and child abuse on lifetime diagnosis of major depression; however, the distribution of the protective alleles in the different groups was consistent with an over-representation of the protective *CRHR1* alleles in individuals with child abuse but no lifetime major depression. This analysis was further limited by a small sample size, prohibiting analysis with a more graded child abuse variable due to very small Ns/cell (see Table IV).

The main finding of this manuscript is that the 5-HTTLPR interacts with *CRHR1* variants and child abuse to predict adult depressive symptoms. In fact, the protective effects of the *CRHR1* TCA haplotype were only observed in individuals carrying the 5-HTTLPR LL genotype. On the other hand, the 5-HTTLPR S allele appeared to enhance the effects of lower levels of child abuse only in the additional presence of the *CRHR1* risk haplotypes. Overall, individuals carrying the risk alleles in both genes exhibit more severe depressive symptoms already at lower “doses” of child abuse than individuals with no or only one of the risk alleles (see Fig. 3B). The finding that this interaction is present when child abuse is divided across three levels but not across two levels is consistent with research suggesting a dose–response relationship between the number of types of adverse experiences in early life and increased risk for a number of health and behavioral problems, including major depression, over the lifespan [Edwards et al., 2003; Chapman et al., 2004; Anda et al., 2006]. Note, however, that with sufficient levels of childhood abuse, any amount of genetic “protection” may be overcome.

Although this G × G × E interaction needs further independent replication to be confirmed, it is well in agreement with the previously reported interactions between the serotonergic and CRF system on a cellular and systems level as described in the introduction [De Bellis et al., 1993; Price et al., 1998; Rainnie, 1999; Kirby et al., 2000, 2008; Price and Lucki, 2001; Nikisch et al., 2005; Lowry et al., 2008]. This G × G interaction could also help to clarify the meaning of G × E interaction findings with the 5-HTTLPR or *CRHR1* variants alone. In fact, different frequencies in the combination of 5-HTTLPR and *CRHR1* risk or protective variants could alter the effect size of the individual G × E interaction, if the second variant is not considered. Likewise, our finding that this interaction is present when child abuse is divided across three levels but not across two-level points to the importance of paying attention to the ways in which broad constructs such as “stressful life events” or even more specific constructs such as “child abuse” are defined in evaluating the meaning of G × E research. Within these broad categories are varying stressful experiences for which the underlying neurobiological processes contributing to risk/resilience are likely to be different.

Our study has several limitations such as the use of a non-diagnostic, self-report measure of current depression symptoms for the main analysis. A subset of our data now has lifetime and current psychiatric diagnoses according to the SCID-DSMIV [First et al., 1995] and we have previously reported good agreement of BDI scores >16 and current major depressive episode [see Bradley et al., 2008]. Our interaction results might thus be extended at least to the presence of a current major depressive episode. Unfortunately, the sample with data on current major depression does not have sufficient power yet for meaningful G × E and even less for G × G × E interaction analyses. Likewise, our sample with data on lifetime diagnosis of major depression is still being collected and our G × E interaction analyses using this data should be considered preliminary. As with diagnosis of current depression, our current sample size for lifetime diagnosis of depression does not allow for G × G × E analyses. As with the majority of G × E studies to date, our data are cross-sectional and rely on adult self-reported history of childhood abuse and are thus likely confounded to a certain degree by recall bias. The fact that our data are gathered in sample of low income, African-American men and women presenting for primary care in public hospital also limits the generalizability of the results. However, this limitation is mitigated by the facts that exposure to traumatic events including childhood abuse occurs at a notably higher rate among low income, urban dwelling African-American populations [Alim et al., 2006; Bierut et al., 2007] with a high percentage of this exposure occurring during youth [Shakoor and Chalmers, 1991; Fitzpatrick, 1993]. This level of trauma exposure is associated with increased levels of risk for physical and mental health problems and associated healthcare costs [Fullilove et al., 1998; Schwartz et al., 2005, 2006]. Additionally, the limitation of self-report of history of abuse is a well-known one, which is important to attend to, but which does not alter the primary findings. Certainly, prospective studies are indicated in the future. Understanding the complex relationship between risk and resilience for disease among adults with a history of childhood abuse, particularly in highly traumatized high-risk populations is of high public health relevance.

By design, G × G × E interactions require large sample sizes, so that independent replication and extension in a larger sample will be needed to confirm our finding. Nonetheless, our sample represents one of the larger samples available for G × E interaction analyses and suggests that more complex analyses than case–control associations are necessary to identify the genetic variants relevant in the genetics of depression.
